# Muay Thai–specific high-intensity interval training enhances anaerobic power in elite fighters

**DOI:** 10.3389/fspor.2026.1831605

**Published:** 2026-05-20

**Authors:** Raja Syed Tengku Sulaiman

**Affiliations:** Major in Physical Education, School of Education, Walailak University, Nakhon Si Thammarat, Thailand

**Keywords:** aerobic capacity, anaerobic power, elite fighters, high-intensity interval training, Muay Thai, sport-specific training

## Abstract

**Background:**

Muay Thai requires repeated high-intensity striking efforts supported by both anaerobic and aerobic energy systems. However, evidence regarding the effectiveness of Muay Thai-specific high-intensity interval training (HIIT) protocols in elite fighters remains limited. This study investigated whether a short-term Muay Thai-specific HIIT program could enhance anaerobic power while preserving aerobic adaptations comparable to traditional training.

**Methods:**

Twenty elite male Muay Thai athletes aged 18-28 years were randomly assigned to either a HIIT group (*n* = 10) or a traditional-training control group (CON; *n* = 10). The HIIT protocol combined weighted-glove shadowboxing with explosive sandbag kicking drills, whereas CON maintained their habitual training. Anaerobic performance was assessed using a 30-s Wingate Anaerobic Test, and aerobic capacity was estimated using a 20-m shuttle run test.

**Results:**

Compared with CON, the HIIT group demonstrated significant improvements in anaerobic performance, including peak power (Δ = +1.09 W·kg⁻¹; g = 3.59), mean power (Δ = +0.52 W·kg⁻¹; g = 2.08), and minimum power (Δ = +0.62 W·kg⁻¹; g = 2.44), with significant group × time interactions observed for all outcomes (all *p* < 0.001). Aerobic capacity increased over time in both groups (*p* < 0.001), with no significant group × time interaction (*p* = 0.208), indicating comparable aerobic adaptations between groups.

**Conclusion:**

Muay Thai-specific HIIT substantially improved anaerobic power while maintaining aerobic adaptations comparable to traditional training. These findings support the use of sport-specific HIIT as a practical and time-efficient conditioning strategy for elite combat-sport athletes.

## 1 Introduction

Muay Thai is a high-intensity striking combat sport characterized by repeated explosive exchanges of punches, elbows, knees, and kicks interspersed with brief recovery periods. These exchanges impose substantial metabolic and neuromuscular demands on athletes. Successful performance requires fighters to repeatedly generate high force and velocity while maintaining technical precision and tactical awareness under considerable physiological stress. Accordingly, both aerobic capacity and anaerobic power are critical for sustaining repeated high-intensity actions and maintaining performance across exchanges (1, 2). These capacities are widely recognized as key determinants of striking performance in combat sports (1–3).

High-intensity interval training (HIIT), involving repeated near-maximal efforts separated by short recovery periods, is an effective and time-efficient method for developing both aerobic and anaerobic energy systems. Previous research has shown that HIIT can improve maximal oxygen uptake (VO_2_max), lactate kinetics, neuromuscular performance, and anaerobic power across a range of athletic populations (2–5). In combat sports, HIIT-based conditioning is particularly relevant because the intermittent and explosive nature of competition requires athletes to perform repeated high-intensity actions (2–4).

Recent work in striking-based combat sports such as kickboxing and mixed martial arts (MMA) further supports the use of HIIT to enhance anaerobic power and high-intensity performance. Interventions in kickboxing athletes have been reported to improve repeated striking ability and anaerobic capacity, while studies in MMA fighters describe gains in both anaerobic performance and sport-specific conditioning. Together, these findings underscore the relevance of high-intensity, intermittent training for combat sports that involve repeated explosive actions and variable recovery patterns, and they highlight shared physiological demands across striking-based disciplines.

Despite the growing use of HIIT for combat-sport conditioning, evidence on sport-specific HIIT protocols in traditional Southeast Asian striking sports such as Muay Thai remains limited. Systematic reviews and meta-analyses indicate that HIIT can enhance aerobic capacity and anaerobic power in Olympic combat sports (2, 6–8). Technique-specific interval protocols in disciplines such as taekwondo and judo have been shown to improve Wingate-derived anaerobic performance (6–8), and studies in elite Wushu athletes report concurrent increases in anaerobic capacity and maximal oxygen uptake (9, 10). In the context of elite Muay Thai, these findings provide a physiological rationale to expect comparable adaptations when training incorporates discipline-specific movement patterns.

However, controlled intervention studies evaluating Muay Thai–specific HIIT in elite fighters are still scarce. Conditioning programs that integrate authentic striking movements may enhance ecological validity while directly targeting performance-relevant physiological capacities. In particular, combining upper- and lower-limb explosive actions within supramaximal interval structures may offer a practical means of improving both anaerobic power and aerobic adaptations.

Therefore, this study examined the effects of a Muay Thai–specific HIIT program that combined weighted-glove shadowboxing with explosive sandbag roundhouse kicking on Wingate-derived anaerobic power and estimated aerobic capacity in elite fighters. We hypothesized that Muay Thai–specific HIIT would produce greater improvements in anaerobic power than traditional training while eliciting comparable changes in aerobic capacity (2, 10, 11).

## 2 Materials and methods

### 2.1 Study design

A parallel-group randomized controlled trial was conducted to examine the effects of a Muay Thai–specific high-intensity interval training (HIIT) program on estimated aerobic capacity and Wingate-derived anaerobic power in elite Muay Thai athletes, two key physiological determinants of combat-sport performance. Laboratory- and field-based assessments (30-s Wingate test and 20-m shuttle run) were selected to reflect the intermittent, high-intensity demands of competitive Muay Thai and to quantify training-induced changes in anaerobic performance and estimated maximal oxygen uptake (VO_2_max) (12–14). Participants were randomly allocated to either a Muay Thai–specific HIIT group (HIIT) or a traditional-training control group (CON). All pre- and post-intervention assessments were conducted under standardized conditions.

### 2.2 Participants

Twenty elite male Muay Thai athletes (aged 18–28 years) competing at the national level, each achieving at least silver-medal status for two consecutive years, volunteered to participate in the study. An *a priori* sample size calculation was performed using Cohen's method (*α* = 0.05, power = 0.80, expected effect size = 0.60), indicating that a total sample of 20 participants would be sufficient to detect between-group differences (15). From an initial pool of 44 athletes assessed for eligibility, 20 were randomly allocated to either the Muay Thai–specific HIIT group (*n* = 10) or the control group (CON; *n* = 10).

The remaining athletes were excluded due to scheduling conflicts with upcoming competitions, minor injuries or medical conditions identified during screening that contraindicated high-intensity training, or logistical constraints (e.g., travel distance or inability to commit to the full two-week protocol). Only participants who completed the intervention and all testing procedures were included in the final analysis.

**Figure 1:**
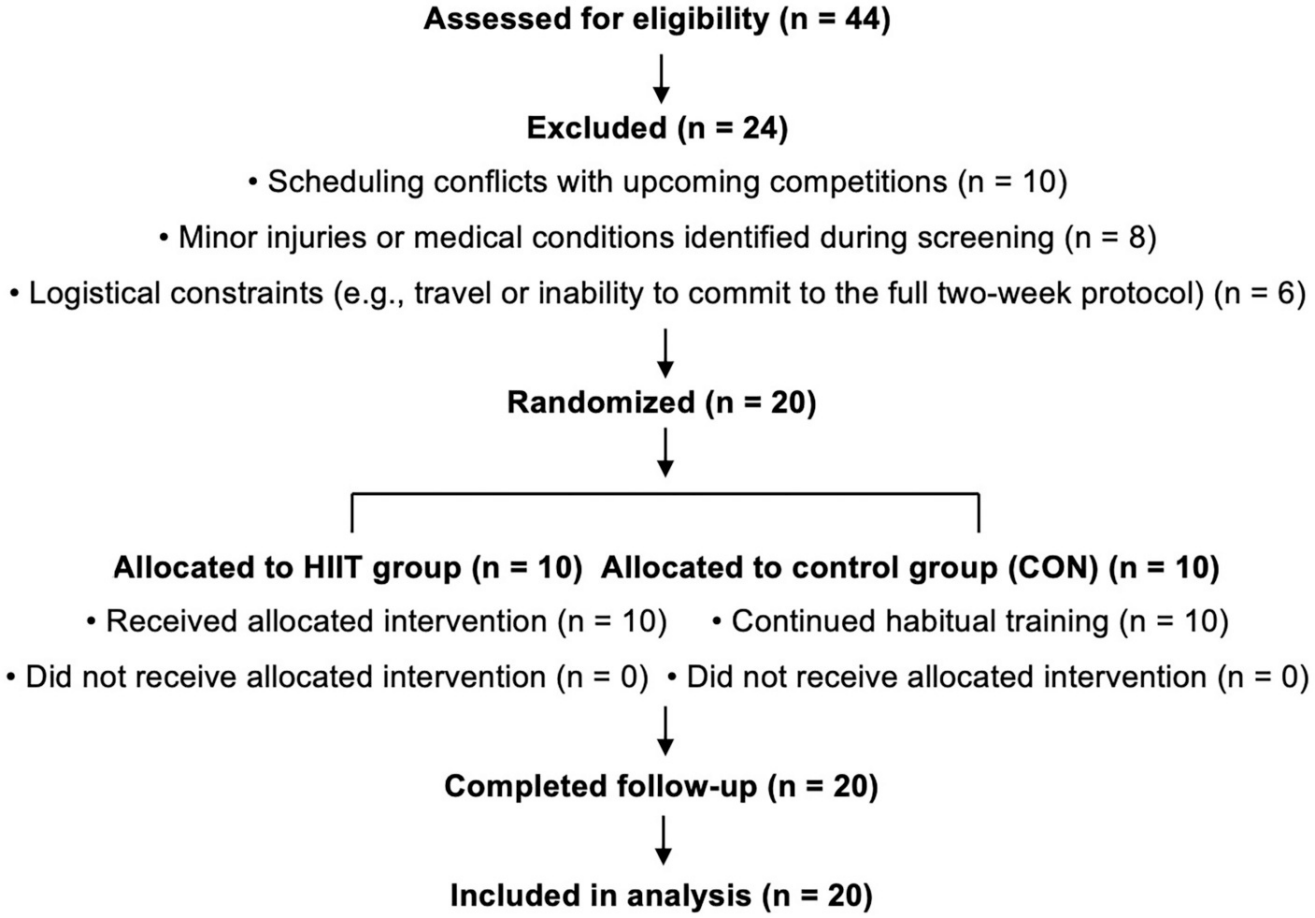
CONSORT-style flow diagram of participant recruitment, screening, randomization, allocation, follow-up, and analysis. The diagram summarizes the number of athletes assessed for eligibility, excluded (with reasons), allocated to each group, and included in the final analysis.

Participant flow, including recruitment, screening, randomization, allocation, follow-up, and analysis, is presented in a CONSORT-style flow diagram (Figure 1).

All procedures were conducted in accordance with the Declaration of Helsinki and were approved by the Human Research Ethics Committee of Walailak University (Approval No. WUEC-25-128-01). Written informed consent was obtained from all participants prior to data collection.

### 2.3 Aerobic performance assessment

Aerobic capacity was assessed using the 20-m shuttle run test, with VO_2_max estimated using the equation of Léger et al. (12). Participants performed repeated 20-m shuttle runs paced by progressive audio signals until volitional exhaustion, and the final completed stage was used to estimate VO_2_max. This field-based protocol provides a practical and widely used indirect estimate of aerobic capacity but does not directly measure oxygen uptake.

All tests were performed indoors on a flat, non-slip surface with standardized cone placement and the original pacing audio (12, 14, 16, 17).

### 2.4 Training intervention

The Muay Thai–specific HIIT protocol consisted of repeated 30-s supramaximal efforts based on Wingate principles (2, 8, 18). Each 30-s interval comprised two consecutive 15-s sport-specific drills:

1. **Weighted-glove shadowboxing (15 s):** maximal-effort punching while wearing 0.5–1.0 kg gloves to increase neuromuscular activation and punching velocity under fatigue (19, 20).
2. **Explosive sandbag roundhouse kicking (15 s):** continuous maximal roundhouse kicks against a heavy sandbag to target lower-limb anaerobic power and fatigue tolerance (21, 22).

Each HIIT session involved repeated 30-s supramaximal efforts interspersed with 30-s passive recovery periods, yielding a 1:1 work-to-rest ratio. A total of 10 bouts were performed per session, corresponding to 5 min of high-intensity work. Training sessions were conducted three times per week over a two-week intervention period (six sessions in total). Including recovery intervals, the HIIT component lasted approximately 10 min per session.

The two-week intervention was designed to reflect a practical, intensified pre-competition training phase commonly used in Muay Thai, in which high-intensity, sport-specific stimuli are emphasized while overall training volume is relatively reduced. This design enhances ecological validity by aligning the protocol with real-world practices in elite fighters.

All HIIT sessions were supervised by experienced coaches to ensure maximal effort and adherence to the protocol. The control group (CON) continued their habitual training without any additional structured HIIT.

Habitual training in the control group consisted of a combination of technical, tactical, and conditioning sessions typical of elite Muay Thai preparation. These sessions generally included pad work, bag work, sparring, clinch training, and general strength and conditioning exercises. Athletes trained 5–6 times per week, with sessions typically lasting 60–120 min depending on the training focus. Conditioning components often involved moderate- to high-intensity activities such as running, circuit training, and sport-specific drills, reflecting standard practice in elite Muay Thai training environments.

Outcome measures were obtained 72 h before and 48 h after the intervention to minimize residual fatigue and avoid interference with concurrent strength and skill training (10, 23).

Internal training load was not objectively monitored during the intervention. Measures such as heart rate (HR), rating of perceived exertion (RPE), and blood lactate concentration were not collected due to practical constraints associated with field-based implementation in an elite training environment. Consequently, the intensity of the training stimuli cannot be precisely quantified or directly compared between groups.

External variables, including overall training load, sleep, recovery strategies, and nutritional intake, were not standardized or systematically monitored during the intervention period. Participants were instructed to maintain their usual training routines and lifestyle habits; however, adherence to these factors was not formally controlled.

Similarly, the exact content, intensity, and distribution of training components in the control group were not standardized or systematically monitored (e.g., session duration, intensity, or RPE). As a result, potential differences in total high-intensity training volume between groups cannot be excluded, and the present design does not allow a clear distinction between the effects of training specificity and overall training load.

The HIIT protocol was delivered with a consistent structure across sessions to standardize the training stimulus. Participants were instructed to perform each bout at maximal effort, and strong verbal encouragement was provided to help maintain intensity throughout all intervals.

### 2.5 Outcome measures

#### 2.5.1 Aerobic capacity

Estimated VO_2_max (mL·kg^−1^·min^−1^) was calculated as:

$$VO_{2}\max=31.025\;+\;(3.238\times \mathrm{speed})-(3.248\times \mathrm{age})+(0.1536\times \mathrm{speed}\times \mathrm{age})$$

where speed is the final running velocity (km·h^−1^) attained during the 20-m shuttle run (12).

#### 2.5.2 Anaerobic power

Anaerobic performance was assessed using a 30-s Wingate test on a Monark 828E cycle ergometer. Resistance was set at 0.067 × body mass (kg). Peak power, mean power, minimum power (W·kg^−1^), and fatigue index (%) were calculated using standard equations (13, 22). Fatigue index was calculated as:

Fatigueindex=[(peakpower−lowestpower)/peakpower]×100.

### 2.6 Statistical analyses

Data were analyzed using SPSS (version 29.0; IBM Corp., Armonk, NY, USA). Descriptive data are presented as mean ± SD. Normality and homogeneity of variance were assessed using the Shapiro–Wilk and Levene's tests, respectively. A two-way mixed-model ANOVA (group × time) was used to examine main and interaction effects. When significant interactions were detected, Bonferroni-adjusted *post hoc* tests were applied. Statistical significance was set at *p* < 0.05.

Effect sizes were reported as partial eta squared (*η*p^2^) for ANOVA outcomes and Cohen's d for within-group changes and were interpreted according to established conventions in behavioral and sports science research (15, 16). Between-group effect sizes (Cohen's d) were calculated as the difference in mean change scores divided by the pooled standard deviation of the baseline values, and Hedges' g was additionally computed as a bias-corrected estimate to account for potential small-sample bias.

The *a priori* sample size calculation was designed to ensure adequate statistical power to detect within-group changes over time rather than to provide precise population-level estimates of effect size. Accordingly, the standardized effect sizes reported in this study should be interpreted with caution, particularly given the small sample size, the elite and relatively homogeneous nature of the cohort, and the potential for reduced between-subject variability to inflate standardized effect-size estimates.

## 3 Results

### 3.1 Baseline characteristics

No significant baseline differences were observed between the HIIT (*n* = 10) and control (CON; *n* = 10) groups for age, body mass, height, body mass index, body fat percentage, training age, or number of fights (all *p* ≥ 0.16; Table 1).

**Table 1:** Baseline characteristics of the HIIT and control (CON) groups.

Variable	HIIT (*n* = 10) mean ± SD	CON (*n* = 10) mean ± SD	*P* value
Age (years)	22.50 ± 2.64	21.00 ± 3.62	0.305
Body mass (kg)	55.82 ± 2.63	58.59 ± 5.34	0.164
Height (cm)	168.10 ± 4.12	169.50 ± 4.88	0.497
Body mass index (kg·m^−2^)	19.74 ± 1.25	20.35 ± 1.85	0.396
Body fat (%)	12.45 ± 1.89	12.37 ± 2.35	0.927
Training age (years)	5.50 ± 1.96	4.70 ± 2.06	0.385
Fights (*n*)	25.70 ± 5.77	27.60 ± 6.77	0.508

Values are presented as mean ± SD. Between-group differences were assessed using independent-samples *t* tests.

### 3.2 Anaerobic power

**Figure 2:**
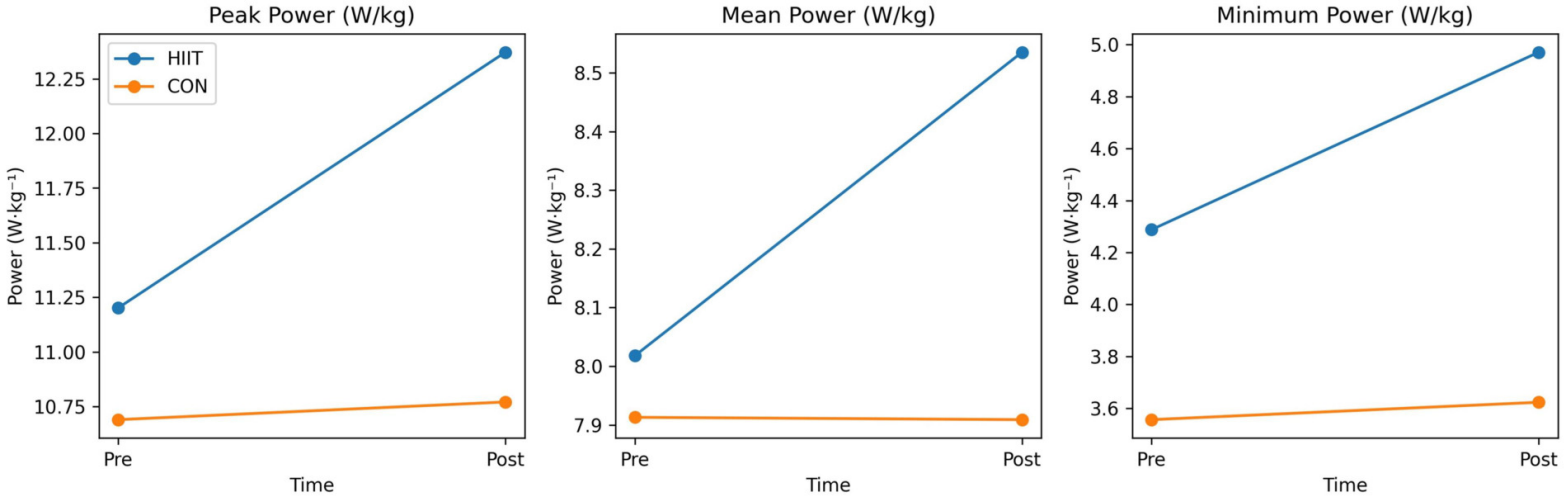
Changes in Wingate-derived anaerobic power following the intervention. Panels show pre- and post-intervention values for peak power, mean power, and minimum power in the Muay Thai–specific high-intensity interval training (HIIT) and control (CON) groups. Lines represent group means across time points and illustrate the group × time interactions for anaerobic power variables.

**Figure 3:**
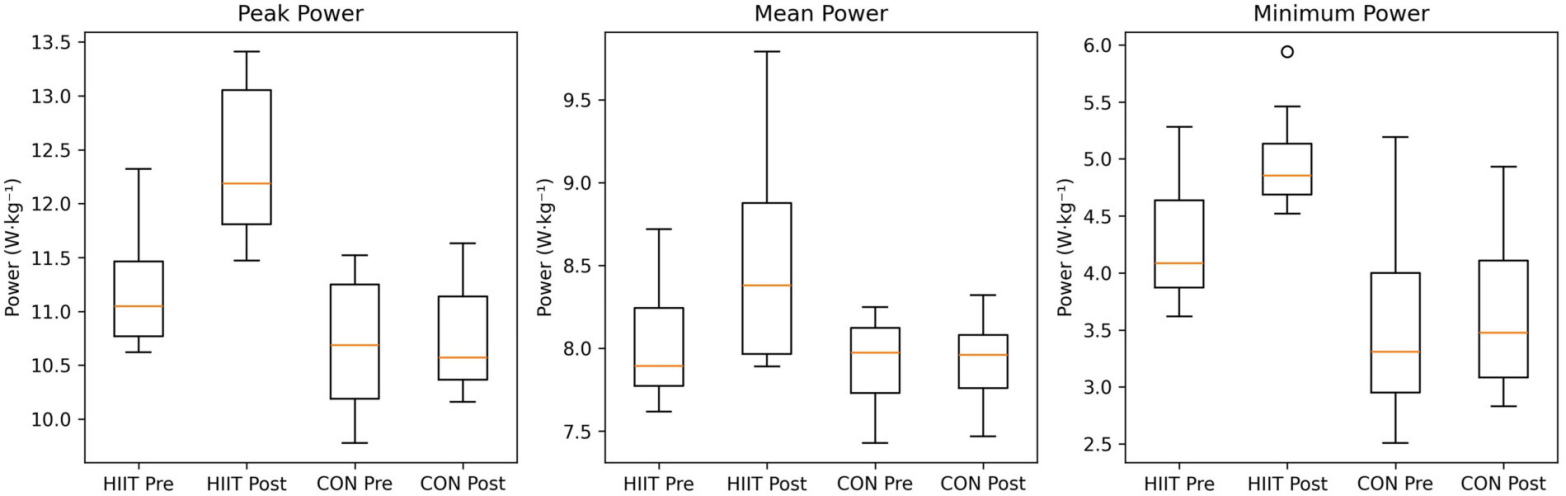
Distributions of Wingate-derived anaerobic power before and after the intervention. Three-panel boxplots display peak power, mean power, and minimum power in the Muay Thai–specific HIIT and CON groups at pre- and post-intervention. Boxes represent interquartile ranges with median lines, whiskers indicate the data range, and points beyond the whiskers denote potential outliers.

Significant group × time interactions were observed for Wingate peak power, mean power, and minimum power (all *p* < 0.001; Table 2; Figures 2, 3).

**Table 2:** Changes in anaerobic power and estimated aerobic capacity following the Muay Thai–specific HIIT intervention in the HIIT and control (CON) groups.

Variable	HIIT Pre	HIIT Post	CON Pre	CON Post	Δ Difference	95% CI	Effect size (Hedges g)	*p* (time)	*p* (group × time)
Wingate peak power (W·kg^−1^)	11.20 ± 0.56	12.37 ± 0.73	10.69 ± 0.64	10.77 ± 0.54	1.09	[0.83, 1.34]	3.59	<0.001	<0.001
Wingate mean power (W·kg^−1^)	8.02 ± 0.36	8.54 ± 0.68	7.91 ± 0.29	7.91 ± 0.27	0.52	[0.31, 0.73]	2.08	0.004	<0.001
Wingate minimum power (W·kg^−1^)	4.29 ± 0.59	4.97 ± 0.45	3.56 ± 0.88	3.62 ± 0.69	0.62	[0.40, 0.83]	2.44	<0.001	<0.001
Fatigue index (%)	69.53 ± 5.53	78.04 ± 3.28	70.11 ± 5.84	79.02 ± 4.03	−0.39	[−2.65, 1.87]	−0.15	<0.001	0.111
Estimated VO_2_max (mL·kg^−1^·min^−1^)	58.2 ± 3.1	61.5 ± 3.0	57.9 ± 2.8	58.9 ± 3.1	—	—	—	<0.001	0.208

Values are presented as mean ± SD. Δ Difference represents the between-group difference in change scores (HIIT – CON). Effect sizes are reported as Hedges’ g. *p* (time) indicates the main effect of time, and *p* (group × time) represents the group × time interaction obtained from the two-way mixed-model ANOVA. The symbol (—) indicates values not reported due to a non-significant group × time interaction.

Peak power showed a very large interaction effect [F(1,18) = 89.77, *p* < 0.001, *η*p^2^ = 0.833], with a greater increase in the HIIT group than in CON [between-group difference = 1.09 W·kg^−1^; 95% CI (0.83, 1.34)]. Mean power also demonstrated a significant interaction [F(1,18) = 17.15, *p* < 0.001, *η*p^2^ = 0.488; between-group difference = 0.52 W·kg^−1^; 95% CI [0.31, 0.73]], as did minimum power [F(1,18) = 22.20, *p* < 0.001, *η*p^2^ = 0.552; difference = 0.62 W·kg^−1^; 95% CI [0.40, 0.83]]. In elite Muay Thai fighters, these results indicate that the sport-specific HIIT protocol elicited substantially greater improvements in Wingate-derived anaerobic power than traditional training.

### 3.3 Aerobic capacity

**Figure 4:**
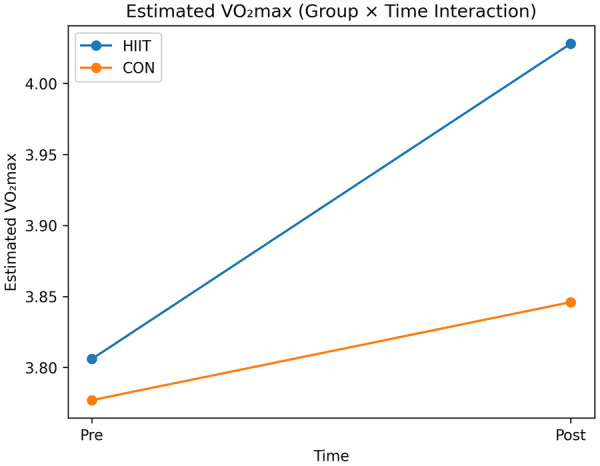
Changes in estimated maximal oxygen uptake (VO_2_max) following the intervention. The interaction plot shows pre- and post-intervention VO_2_max values for the Muay Thai–specific HIIT and CON groups. Lines represent group means across time points. Although the HIIT group showed a numerically greater increase, the group × time interaction was not statistically significant, indicating comparable aerobic adaptations between groups.

For estimated VO_2_max, there was a significant main effect of time (*p* < 0.001), indicating improvements in aerobic capacity across both groups, but no significant group × time interaction [F(1,18) = 1.71, *p* = 0.208, *η*p^2^ = 0.087; Table 2; Figure 4]. The between-group difference in change was small and not statistically significant, indicating comparable improvements in aerobic capacity between groups.

### 3.4 Fatigue index

No significant group × time interaction was found for fatigue index [F(1,18) = 2.80, *p* = 0.111, *η*p^2^ = 0.135; Table 2]. Although fatigue index increased in both groups following the intervention, the confidence interval for the between-group difference crossed zero [−2.32%; 95% CI (−5.24, 0.59)], indicating no meaningful difference between the HIIT and CON groups.

## 4 Discussion

### 4.1 Summary of main findings

The present study shows that a Muay Thai–specific high-intensity interval training (HIIT) protocol can improve Wingate-derived anaerobic power within a short, two-week training period in elite fighters. Significant group × time interactions for peak, mean, and minimum power indicate that the sport-specific HIIT stimulus was associated with greater gains in anaerobic performance than habitual training. However, because training load in the control group was not standardized or systematically monitored, these findings cannot be interpreted as definitive evidence that the protocol is superior to a volume-matched high-intensity program. The absence of an active, volume- and intensity-matched control condition further limits the ability to isolate the independent effect of training specificity.

Estimated VO_2_max improved over time in both groups without a significant interaction effect, indicating comparable aerobic adaptations. These findings suggest that aerobic improvements were likely driven by overall exposure to high-intensity training rather than by the specific structure of the HIIT protocol. Collectively, the results indicate that integrating authentic striking movements into supramaximal interval structures may represent a time-efficient strategy for enhancing neuromuscular and metabolic qualities that underpin explosive performance in combat sports (1, 2, 6–9, 11, 18, 24, 25).

Importantly, improvements in laboratory-based measures of anaerobic power (e.g., the Wingate test) do not necessarily translate directly into enhanced sport-specific performance. Because the present study did not include biomechanical or technical assessments (e.g., striking velocity, impact force, or accuracy under fatigue), a direct causal link between anaerobic power gains and in-ring performance cannot be established. The two-week intervention was designed to reflect a pre-competition “taper-plus-sharpening” phase commonly used in Muay Thai, during which high-intensity, sport-specific stimuli are emphasized while overall training volume is reduced. Accordingly, the present findings should be interpreted as evidence of short-term responsiveness rather than long-term adaptation.

### 4.2 Anaerobic adaptations to Muay Thai–specific HIIT

The improvements in Wingate-derived power likely reflect rapid neuromuscular and metabolic adaptations induced by repeated supramaximal efforts performed within sport-specific movement patterns. Weighted-glove shadowboxing may increase upper-limb loading and neuromuscular activation, whereas explosive sandbag roundhouse kicking likely imposes substantial demands on lower-limb force production and rate of force development (19–22). Together, these stimuli may enhance motor-unit recruitment, phosphocreatine resynthesis, and intramuscular energy transfer, thereby improving short-duration maximal power output. This interpretation aligns with evidence that HIIT enhances anaerobic performance through neuromuscular and metabolic pathways across athletic populations (2–5, 24–26).

The present findings are consistent with previous studies in kickboxing and mixed martial arts (MMA), which have reported that high-intensity interval training can substantially improve anaerobic performance and repeated high-intensity actions. As in these sports, Muay Thai involves intermittent explosive efforts interspersed with brief recovery periods, suggesting that similar physiological adaptations may underpin performance improvements across striking-based combat disciplines. The current study extends this literature by implementing a fully sport-specific HIIT protocol based on authentic Muay Thai striking movements, thereby enhancing ecological validity and strengthening the potential transfer of physiological adaptations to real-world performance contexts.

In striking combat sports, where performance depends on repeated explosive actions, such adaptations may contribute to improved striking performance and fatigue tolerance during high-intensity exchanges. Specifically, enhanced force production capacity and a greater ability to sustain repeated high-intensity efforts are likely to be key determinants of performance under competitive conditions (1, 19–23). From a performance perspective, increases in anaerobic power may translate to more effective striking in Muay Thai, particularly in the ability to generate higher force and velocity during repeated offensive actions. Given that competitive exchanges involve successive high-intensity strikes with limited recovery, improvements in short-duration power output may support greater striking intensity and the capacity to sustain offensive output across rounds.

Fatigue index did not differ significantly between groups, indicating that the Muay Thai–specific HIIT protocol did not meaningfully alter fatigue resistance compared with traditional training. The increase in fatigue index observed in both groups is likely attributable to higher peak power output during the Wingate test, which increases the relative decline in power across the 30-s effort. Athletes who generate greater peak power may therefore exhibit a larger proportional reduction in power, a pattern commonly observed in supramaximal anaerobic testing. Accordingly, the observed increase in fatigue index should be interpreted in the context of enhanced maximal power output rather than as evidence of reduced fatigue resistance.

### 4.3 Considerations for effect-size interpretation

Although the observed changes were substantial, the magnitude of the standardized effect sizes should be interpreted with caution. The relatively small and homogeneous sample of elite fighters likely reduced between-subject variability, which can inflate standardized indices such as Cohen's d and Hedges' g even when absolute changes are moderate (15). In addition, the large within-group improvements observed over a short intervention period may have further contributed to elevated effect-size estimates.

Similar patterns have been reported in high-intensity training studies involving well-trained cohorts, where restricted baseline variability and strong responsiveness to targeted stimuli yield disproportionately large standardized effects (24–32). Accordingly, the present effect sizes should be considered alongside absolute performance changes rather than as precise estimates of population-level magnitude. Replication in larger and more diverse samples is therefore required to confirm the robustness and generalizability of these findings.

### 4.4 Aerobic adaptations and comparison with previous research

The significant main effect of time for estimated VO_2_max indicates that both training approaches were sufficient to stimulate aerobic adaptation over the two-week period. However, the absence of a significant group × time interaction suggests that these improvements were not uniquely attributable to the Muay Thai–specific HIIT protocol but were more likely driven by overall exposure to high-intensity training. This interpretation is consistent with previous research showing that multiple high-intensity training modalities can elicit comparable improvements in VO_2_max in trained athletes (2, 18, 24, 25, 30–36). It also highlights the potential influence of overall training load, which was not controlled across groups and may have contributed to the similar aerobic adaptations observed.

The magnitude of VO_2_max change in the present study appears modest and toward the lower end of the range typically reported in HIIT meta-analyses involving trained populations, which often include longer interventions and more tightly controlled training loads (24, 25, 30–36). This suggests that, in already well-conditioned combat-sport athletes, short-term Muay Thai–specific HIIT may contribute to aerobic adaptations but is unlikely to provide a clear additional advantage over other high-intensity conditioning formats when total training load is comparable. Taken together, the findings support the view that multiple forms of high-intensity training can produce similar aerobic adaptations when the overall training stimulus is sufficient.

### 4.5 Practical applications for Muay Thai conditioning

Integrating discipline-specific high-intensity interval training (HIIT) into technical training sessions may offer a time-efficient approach to improving anaerobic power without substantially increasing overall training volume. This is particularly relevant in combat sports such as Muay Thai, where coaches must balance technical and tactical preparation with physiological conditioning within limited training time.

Evidence from taekwondo, judo, and Wushu suggests that technique-based interval protocols can enhance anaerobic capacity and sport-specific performance (6–11, 18, 20, 21, 24–26, 29). In the context of elite Muay Thai, these findings support the use of conditioning protocols centered on authentic striking drills, which may help bridge the gap between physiological development and technical execution in high-performance environments.

In practical terms, improvements in anaerobic power may enhance key performance attributes in Muay Thai, including striking force, combination speed, and the ability to maintain high-intensity output during repeated exchanges. These adaptations are highly relevant in competitive contexts, where the capacity to sustain powerful strikes under fatigue may influence scoring effectiveness and competitive success. Accordingly, incorporating sport-specific HIIT into training programs may provide a targeted strategy to improve both physiological capacity and its transfer to performance-relevant actions in elite fighters.

Nevertheless, these physiological adaptations represent indirect indicators of performance rather than direct measures of competitive success. While improvements in anaerobic power are likely relevant to Muay Thai performance, their translation to actual competitive outcomes remains to be confirmed. Future research should therefore include direct performance-based assessments (e.g., striking velocity, impact force, and competition-specific metrics) to establish the functional significance of these adaptations in real-world settings.

### 4.6 Methodological considerations and future directions

Several limitations should be considered when interpreting the present findings. First, the relatively small sample of elite male fighters may limit generalizability and contribute to uncertainty in effect estimates, underscoring the need for larger, multicentre trials (15, 25, 26, 33). Second, the two-week intervention period limits conclusions regarding long-term adaptations or the persistence of the observed effects. The findings should therefore be viewed as reflecting short-term responsiveness to a high-intensity, sport-specific training stimulus rather than sustained physiological adaptation. Longer interventions are needed to determine whether these adaptations are maintained, augmented, or attenuated over time. Third, the sample comprised only elite male athletes from a single training center, which further constrains external validity.

Fourth, estimated VO_2_max was derived from the 20-m shuttle run rather than measured directly via gas analysis, which may introduce measurement error and limit precision at the individual level (12, 16, 17). Although this method is widely used in applied sport settings, it provides only an indirect estimate of aerobic capacity. Future studies should incorporate direct cardiopulmonary exercise testing (e.g., breath-by-breath gas analysis) to obtain more accurate and sensitive measures of aerobic adaptation.

Furthermore, internal training load was not objectively monitored using physiological or perceptual measures such as heart rate, rating of perceived exertion, or blood lactate concentration. The absence of these measures limits the ability to quantify exercise intensity and to verify whether training stimuli were equivalent or systematically different between groups. Future research should include objective and subjective monitoring of internal load to improve control of training intensity and strengthen causal interpretation.

External factors such as overall training load, sleep, recovery, and nutritional intake were not controlled or systematically monitored. Variability in these factors may have influenced individual responses to the intervention and therefore cannot be excluded as potential confounders. Standardized monitoring or control of these variables in future studies would improve internal validity and strengthen causal inference.

Importantly, sport-specific performance outcomes (e.g., striking velocity, impact force, or competitive performance) were not directly assessed. Thus, the observed improvements should be interpreted as enhancements in laboratory-based physiological markers rather than confirmed improvements in competitive performance. Although these markers are theoretically linked to performance, the extent to which they translate into actual competitive advantage remains to be determined.

Finally, the control group maintained habitual training rather than an active, volume- and intensity-matched comparator, and training load was not systematically monitored. As a result, the independent effects of the Muay Thai–specific HIIT protocol cannot be clearly separated from those attributable to differences in overall training volume or intensity. The present findings should therefore be interpreted as reflecting the effectiveness of a structured HIIT block relative to unstandardized habitual training, rather than a controlled comparison under matched training loads.

Future research should incorporate volume- and intensity-matched comparator interventions, standardized training-load monitoring, and a broader range of performance outcomes (e.g., striking velocity, impact force, neuromuscular fatigue). In addition, integrating detailed biomechanical and technical assessments would help clarify whether improvements in laboratory-derived anaerobic power translate into meaningful sport-specific performance gains.

## 5 Conclusion

The present findings indicate that a short-term, Muay Thai–specific high-intensity interval training (HIIT) program can improve anaerobic power in elite fighters over a two-week period. Significant increases in Wingate-derived peak, mean, and minimum power suggest that integrating sport-specific striking drills within supramaximal interval structures effectively stimulates neuromuscular and metabolic adaptations relevant to combat-sport performance, likely through enhanced motor-unit recruitment, rate of force development, and phosphagen system contribution.

Estimated aerobic capacity improved over time in both groups; however, these changes were not uniquely attributable to the HIIT protocol. This pattern suggests that, in well-conditioned fighters, short-term sport-specific HIIT does not provide a clear additional aerobic advantage over other high-intensity conditioning formats, and that aerobic adaptations are more strongly influenced by overall exposure to high-intensity training.

Taken together, the results support the use of Muay Thai–specific HIIT as a practical and time-efficient conditioning strategy for enhancing explosive performance capabilities in elite combat-sport athletes. From an applied standpoint, incorporating discipline-specific HIIT into training programs may optimize anaerobic development without substantially increasing overall training load, whereas improvements in aerobic capacity are likely governed more by total high-intensity training stimulus than by the precise conditioning format, particularly in athletes with already elevated baseline fitness.

## Data Availability

The raw data supporting the conclusions of this article will be made available by the authors, without undue reservation.
